# Preliminary Investigation on Phytoplankton Dynamics and Primary Production Models in an Oligotrophic Lake from Remote Sensing Measurements

**DOI:** 10.3390/s21155072

**Published:** 2021-07-27

**Authors:** Ilaria Cesana, Mariano Bresciani, Sergio Cogliati, Claudia Giardino, Remika Gupana, Dario Manca, Stefano Santabarbara, Monica Pinardi, Martina Austoni, Andrea Lami, Roberto Colombo

**Affiliations:** 1 Remote Sensing of Environmental Dynamics Laboratory, DISAT, University of Milano-Bicocca, Piazza della Scienza 1, 20126 Milan, Italy; sergio.cogliati@unimib.it (S.C.); roberto.colombo@unimib.it (R.C.); 2 Institute of Electromagnetic Sensing of the Environment, National Research Council of Italy (CNR-IREA), via Bassini 15, 20133 Milan, Italy; bresciani.m@irea.cnr.it (M.B.); giardino.c@irea.cnr.it (C.G.); pinardi.m@irea.cnr.it (M.P.); 3 Eawag Swiss Federal Institute of Aquatic Science & Technology, Surface Waters—Research and Management, Überlandstrasse 133, 8600 Dübendorf, Switzerland; remika.gupana@eawag.ch; 4 Department of Geography, University of Zurich, Winterthurerstrasse 190, 8057 Zurich, Switzerland; 5 Water Research Institute, National Research Council of Italy (CNR-IRSA), Corso Tonolli 50, 28922 Verbania, Italy; dario.manca@cnr.it (D.M.); andrea.lami@irsa.cnr.it (A.L.); 6 Photosynthesis Research Unit, National Research Council of Italy (CNR-IBF), Via Celoria 26, 20133 Milan, Italy; stefano.santabarbara@cnr.it; 7 EcoDyctya Srl, 21020 Varese, Italy; m.austoni@ecodictya.com

**Keywords:** inland waters, hyperspectral measurements, high-frequency measurements, remote sensing, fluorescence, phytoplankton primary production

## Abstract

The aim of this study is to test a series of methods relying on hyperspectral measurements to characterize phytoplankton in clear lake waters. The phytoplankton temporal evolutions were analyzed exploiting remote sensed indices and metrics linked to the amount of light reaching the target (E_PAR_), the chlorophyll-a concentration ([Chl-a]_OC4_) and the fluorescence emission proxy. The latter one evaluated by an adapted version of the Fluorescence Line Height algorithm (F_FLH_). A peculiar trend was observed around the solar noon during the clear sky days. It is characterized by a drop of the F_FLH_ metric and the [Chl-a]_OC4_ index. In addition to remote sensed parameters, water samples were also collected and analyzed to characterize the water body and to evaluate the in-situ fluorescence (F_F_) and absorbed light (F_A_). The relations between the remote sensed quantities and the in-situ values were employed to develop and test several phytoplankton primary production (PP) models. Promising results were achieved replacing the F_A_ by the E_PAR_ or F_FLH_ in the equation evaluating a PP proxy (R^2^ > 0.65). This study represents a preliminary outcome supporting the PP monitoring in inland waters by means of remote sensing-based indices and fluorescence metrics.

## 1. Introduction

Monitoring phytoplankton is fundamental in the global climate warming scenario, to evaluate the trophic status of water bodies and to detect potentially harmful blooms [1]. Furthermore, phytoplankton is involved in the primary production (PP) from which organic matter and energy are obtained by photosynthetic process. Consequently, PP is generally associated with the ecosystems food webs and the global carbon cycle [2]. In this framework, the photosynthetic pigment chlorophyll-a (Chl-a), contained in all phytoplankton taxa, is routinely used as a proxy to infer the phytoplankton biomass and for primary production estimations [3]. Since more than four decades, Chl-a concentration can be estimated by means of remote sensing techniques, allowing a characterization of the aquatic environments [4] at several spatio-temporal scales. Generally, the remote sensing approaches rely on measurements of the upwelling light spectral changes as a consequence of changes of water inherent optical properties and their related compounds [2,5,6].

One of the possible approaches to remotely monitor both aquatic and terrestrial environments/vegetation relies on exploiting the Sun Induced Chl-a Fluorescence (SICF, hereinafter F) [7,8,9,10]. Basically, fluorescence represents one of the four possible dissipation pathways for the absorbed solar light, along with the photochemical utilization by photosynthesis, heat dissipation and triplet population by intersystem crossing [11,12]. For these reasons, F is closely interrelated to the chlorophyll-a concentration [Chl-a], the photosynthetic machinery and then to the environment primary production. Furthermore, the fluorescence is an optical signal, therefore it affects spectrally (emission region from 640 to 850 nm) the rising-from-the-target radiance and can be potentially detected by sensors mounted on different platforms. Even though F evaluation represents a well-established and non-invasive approach to monitor ecosystems at several spatio-temporal scale, its retrieval from proximal and remote sensed measurements is challenging. The fluorescence, indeed, cannot be easily measured: it is a very weak signal and superimposed to the radiance reflected by the target [13]. Therefore, disentangling these two contributions is not trivial. Despite these difficulties, there are several algorithms allowing the F retrieving [13] in which the strategies applied depend on different factors, such as the environments investigated, the platforms considered or the sensors used.

Chl-a fluorescence emission spectra are well defined with theoretically two emission peaks at 685 nm and 740 nm [8]. In the terrestrial vegetation, most of the algorithms retrieve F around few discrete solar or telluric absorptions lines [13], giving reliable results regardless of the scale considered. For example, the Spectral Fitting Method, SFM [14], evaluates F in the two atmospheric oxygen absorption bands, placed, respectively, around 687 nm (O_2_B) and 760 nm (O_2_A). However, more sophisticated methods exploiting contiguous wavebands have been recently develop, but their reliability was only assessed on ground and/or synthetic measurements. An example is described in Cogliati et al. (2019) [15].

Conversely, in the aquatic environments only the first peak dominates the phytoplankton F spectrum while the second peak is almost completely diminished by the strong water absorption [10]. Moreover, the F retrieval can be also affected by the water body constituents and corresponding bio-optical properties [16] which Mobley et al. (2004) practically distinguished in: (i) Case-1 waters, in which the optical properties are determined primarily by phytoplankton and related colored dissolved organic matter (CDOM) and detritus degradation products; and (ii) Case-2 waters where the optical properties are significantly influenced by other constituents such as mineral particles, CDOM or microbubbles, whose concentrations do not covary with the phytoplankton concentration [17]. Oceanic waters usually belong to Case-1 and leading a variety of methods for F estimations, such as the Fluorescence Line Height (FLH) algorithm, for then understating phytoplankton processes (e.g., Behrenfeld et al. (2009) [18]). In the open ocean, FLH determined in the conventional manner usually correlates well with the actual fluorescence amplitude [16]. Unfortunately, this retrieval is much more complicated in Case-2 waters (e.g., coastal waters) where chlorophyll fluorescence overlaps with a strong NIR elastic scattering peak [16]. For this reason, Case-2 waters represent a more challenging case of study due to the active compounds in the medium, in addition to the phytoplankton, affecting the sensed signal [16].

Lakes, that belong to Case-2, are typically characterized by a high degree of spatio-temporal variability of the inherent optical properties and related compounds. At hourly and daily scales, the acquired signal trends are mainly determined by the phytoplankton dynamics, depending on the light and nutrients availability, but also to variations in the suspended matter concentrations linked to wind-induced resuspension of the bottom sediments or to erosion and run-off from the catchment [19], typical of shallow lakes.

Obtaining a valid fluorescence proxy from remotely sensed measurements could be crucial in the lakes monitoring framework because F responds promptly to all the competing photosynthetic processes [20] and therefore can potentially follows the phytoplankton dynamics. Moreover, its link with the photosynthesis can represent a promising approach for estimating the phytoplankton PP in inland waters exploiting remote sensed measurements and related spectral indices. To improve the fluorescence-based approach to lake, we have conceived a one-week experiment on the Lake Maggiore (Italy) in July 2019. Further details about the water body features and the campaign are reported in Section 2.1 and Section 2.2.

Concerning Case-2 waters, in literature there are already examples of remote sensed quantities used to study the PP at a large scale. For instance, Deng et al. (2017) [21], applied the Vertical Generalized Productivity Model (VGPM) on MODIS data to investigate the long-term variations in PP in Lake Taihu (China). Even though good results were obtained with this approach, the method described was not applicable to the Lake Maggiore experiment, because some crucial input were not acquired during the field campaign. Moreover, the VGPM does not exploit the fluorescence signal in its formulation. Considering the fluorescence as a proxy to gain information about the ecosystem PP is a strategy routinely used in the terrestrial vegetation framework. In this regard, the models developed rely on the concept of light use efficiency [22]. Specifically, they state that carbon fixation is considered as a function of the incident photosynthetically active radiation absorbed by vegetation and of the light-use efficiency (ε), representing the conversion efficiency of absorbed energy to fixed carbon [23]. The latter one can be estimated exploiting the remote sensed F signal at leaf scale [24] and at canopy level [25,26]. Nevertheless, this rationale could be opportunely adapted to aquatic environments. According to Kiefer et al. (1989) [27] and Morrison (2003) [28], the in-situ fluorescence (F_F_) and carbon assimilation (F_C_) can be evaluated as the product between the light absorbed by the phytoplankton (F_A_) contained in a unit of water volume and the quantum yield of fluorescence (Φ_F_) and carbon fixation (Φ_C_), respectively:

(1) $$F_{F}=\Phi_{F}*F_{A}$$

(2) $$F_{C}=\Phi_{C}*F_{A}$$

where F_C_, measured in mol cm^−3^ s^−1^, could be considered as a proxy for the water body PP. By combining these two equations, it is possible to obtain F_C_ from F_F_. Even though this approach is feasible, it is limited by the small volumes of water considered. In this sense, remote sensing (RS) techniques represent a more practical and economical strategy to monitor these environments at a larger scale that can be also easily integrated into a geographic information system [29,30]. Nevertheless, the methods need to be implemented to make them appropriate for these ecosystems (i.e., lakes) [31]. This observation, together with the link between F_C_ and F_F_, represented the starting point to develop and test for the first time several phytoplankton PP models parametrized by means of spectral indices and the fluorescence related metric in optically complex waters. However, in this work only a preliminary investigation was possible due to the few field measurements available.

## 2. Materials and Methods

### 2.1. Study Area

Lake Maggiore is a large (surface area = 213 km^2^, volume = 38 km^3^), oligo-mesotrophic water body located at the south of the Alps between Italy (ca. 80%) and Switzerland (ca. 20%). Formed by glacial erosion in a pre-existing fluvial valley, it is characterized by a maximum depth of 370 nm, is considered holo-oligomictic and rarely undergoes to a complete mixing [32]. The lake has 33 tributaries and only one emissary, the River Ticino [33]. Similar to most lakes in Italy and Central Europe, it underwent to anthropogenic eutrophication during the second half of the 20th century [34] with peak of phosphorus (P) loads at mid-seventies [35]. In the following decades, sewage treatment plants were improved and total phosphorus in detergents were reduced, until values close to pre-industrial concentrations were reached [36]. Its ecology, geochemistry and climate have been monitored since the 1970s [37]. Furthermore, a comprehensive long-term dataset of phytoplankton records and environmental variables is available [37]. In general, the phytoplankton communities appear to have been resilient in the recovery phase [38], but major shifts in community structure became evident from the late 1980s [39]. As reported in [37] the response of phytoplankton to weather conditions show a different response for several groups, in particular, rainfall can have a positive effect on the growth of Cyanobacteria. Conversely, the wind drives the mixing regime and the replenishment of nutrients in spring. In this framework, the diatoms reach their maximum growth while higher wind speeds have a negative impact on the growth of Cyanobacteria. Water temperature and light intensity have a strong effect on the growth of *Mougeotia* sp. (Chlorophytes) and Cyanobacteria.

### 2.2. Field Experiment Description

**Figure 1:**
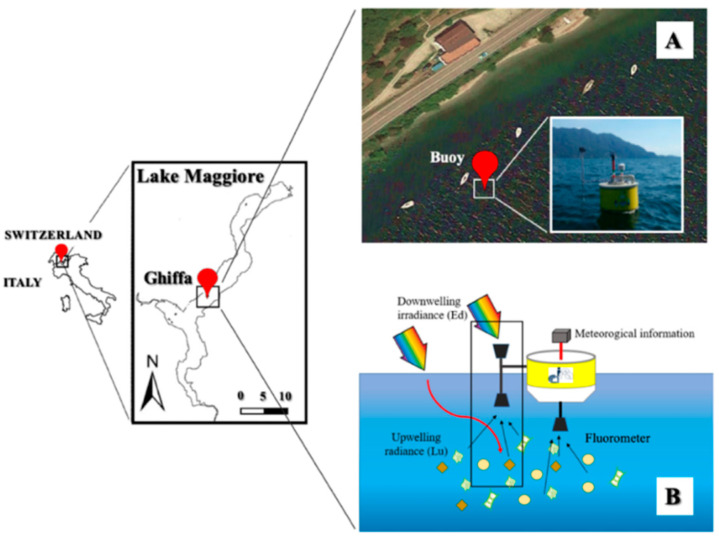
Location of the experimental measurements in the Lake Maggiore. (**A**) (top right) show the buoy on which instruments for hyperspectral and continuous measurements were mounted. (**B**) (bottom right) display the experimental set-up. The water samples have been collected manually close enough to the buoy, to improve the matching between the water samples and the continuous spectral measurements.

The in-situ measurements analyzed in this study have been collected in the Ghiffa site situated on the Lake Maggiore western shore in a week-long campaign from the 2 up to the 7 July 2019 (Figure 1). Continuous hyperspectral acquisitions have been carried out by a ROX spectrometer sensor (JB Hyperspectral devices, Germany) mounted on a floating buoy (Figure 1A). The buoy was set far enough from the coastal zone (around 50 m) to avoid the bottom contribution in the measured spectral signal. The ROX is a field spectrometer designed to acquire continuous spectral measurements in the VNIR region. The instrument employs an Ocean Optics spectroradiometer that collect the incoming and the upwelling irradiance/radiance almost simultaneously in the wavelength interval between 400–950 nm, with a spectral resolution of 1.5 nm and acquisition time of about 1 min. The ROX is equipped with two separated probes, the first one pointed upward to collect the incoming irradiance (E_d_) reaching the target and the second one pointed downward to measure the upwelling radiance (L_u_) rising from the water body, as shown in the black box in Figure 1B. The E_d_ optic was mounted on a goniometer to keep the probe as perpendicular as possible to the water surface regardless the buoy oscillations. To avoid the solar glint, instead, the sensor collecting L_u_ has been placed below the water surface (ca. 15 cm) and pointed downward.

Other sensors have been placed on the buoy, specifically high frequency measurements linked to the chlorophyll concentration have been collected using a fluorometer Cyplops-7F (Turner Design, San Jose, CA, USA). This instrument consists of a compact submersible sensor able to acquire the signal produced by the interaction between an impulse light and the water body compounds. The output signal, measured in Volt, is then expected to be proportional to the chlorophyll concentration (Chl [V]), assuming the fluorescence emission yield constant, or varies within a small interval only. Eventually, wind speed and direction were recorded by meteorological sensors mounted on the top of the buoy.

During the experiment, water samples have been collected at different water depths for optical and chemical laboratory characterization: (i) just below the surface z_0_ (0.2–0.5 m); and (ii) at the Secchi Disk depth z_SD_ (5.5–6 m). Secchi disk, indeed, represents a coarse way to evaluate the underwater light penetration exploiting the visual measure of the water transparency [40]. The water samples have been collected close enough to the buoy, to improve the matching between the water samples and the indices retrieved from the continuous spectral measurements. The sampling has been repeated different times of the day, specifically two samples were coincident to the solar noon and the sunset, respectively, to capture the Lake Maggiore phytoplankton properties under significant variable illumination conditions. These samples, collected on the 2 and 3 of July, have been filtered in-situ with a GF/F glass fiber filters and subsequently analyzed in laboratory. The following nomenclature for samples has been used: S1 and S2 correspond to sampling on the first day, while S3 and S4 to the second one. Odd numbers refer measurements undertaken close to the solar noon, conversely even numbers refer to the afternoon ones. The additional labels, z_0_ and z_SD_ (i.e., S1z_0_ and S1z_SD_), indicate the lake water depths at which the samples have been collected.

In the same days (namely the 2 and 3 of July), a water vertical profile of the downwelling irradiance and upwelling radiance were carried out with the Satlantic radiometer. This instrument is equipped with radiometers able to collect the E_d_ and L_u_ between 350.5–796.5 nm (137 channels, ~3.3 nm) at several depths with an acquisition step of 0.1 m. In the upcast mode, used in this work, the sensor starts collecting the incoming irradiance from the surface up to greater depths.

### 2.3. Laboratory Analysis

As described in Bresciani et al. (2016) [41], photosynthetic pigments for high-performance liquid chromatography (HPLC) analysis were extracted in 90% acetone, overnight in the dark, under nitrogen. The extract obtained was used to quantify the [Chl-a]_HPLC_ (in µg/L), while its derivatives and total carotenoids has been estimated by spectrophotometry [42]. Moreover, individual carotenoids and specific pigments were detected by ion pairing, revers-phase HPLC with an Ultimate 3000 (Thermo Scientific) as described in [43].

TSM (Total Suspended Matters) was obtained gravimetrically [44]. Backscattering was measured using a Hobi Labs Hydroscat-6. The spectral absorption coefficients of phytoplankton (a_phy_) and non-algal particles (a_NAP_) [45] were obtained spectrophotometrically using the filter pad technique [46].

Colored dissolved organic matter (CDOM) was measured spectrophotometrically immediately after filtration by means of a Whatman Nucleopore membrane filters (diameter 47 mm, pore size 0.2 µm). The CDOM absorption coefficient at 440 nm (a_CDOM_(440)) was derived according to Kirk (2011) [47].

Phytoplankton samples were collected and analyzed for the purposes of species identification and cell count under an inverted microscope (400× magnification; [48]).

The maximal Chl-a fluorescence quantum yields (Φ_F_) have been estimated by means of an analysis of the excited state decay relaxation, employing a laboratory-assembled time-correlated single photon counting apparatus, as previously described in Remelli and Santabarbara (2018) [49]. In brief, excitation is provided by a pulsed laser diode (PicoQuant 800B), centered at 632 nm, at a repletion rate of 20 MHz, and an intensity of 1 pJ/pulse. Emitted photons are collected with right-angle geometry, through a monochromator (Jasco, Mod. JT-10, Tokyo, Japan, Japan Hamamatsu Photonics) and multichannel-plate photomultiplier (Hamamatsu, R5916U-51). Acquisition electronic, consisting in Time-to-Amplitude Converter (TAC), Constant-Fraction timing Discriminator (CFD) and multichannel timing analyzer (MCA) are embedded and controlled by PC-mounted acquisition board (Becker and Hinkl, SPC-330, Berlin, Germany). Samples were re-suspended from the sampling-filters, in Bold’s basal growth media, and diluted to an OD equivalent to 0.05 cm^−1^ at 680 nm, before the measurements, placed in 3 mm path-length cuvette. To attain the maximal Chl-a emission yield (Φ_F_max,_ hereinafter Φ_F_), 10 µM of the inhibitor DCMU (3-(3,4-dichlorophenyl)-1,1-dimethylurea) has been added to the samples. The measuring conditions avoid artefacts due to re-absorption of emitted photons. All decay traces were collected at 682 nm (FWHM 3 nm) to obtain at least 2 × 104 counts at the peak channel. Signal are fitted with an iteration-reconvolution routine, accounting for the instrument response function (120 ps) which is measured using a scattering solution (Ludox), and using a linear combination of exponential decay as the model kinetic function, using a laboratory written software, as described in Santabarbara et. al., (2017) [50]. The yield is retrieved from the estimation of the mean decay lifetime starting from the fit parameter ($\tau_{m}=\underset{i}{\sum }A_{i}\tau_{i}^{2}/\underset{i}{\sum }A_{i}\tau_{i}$, where τ_i_ and A_i_ are the lifetimes and associated amplitude, respectively) and using the decay of pure Chl-a dissolved in dry methanol as a reference (monoexponential τ = 4.1 ± 0.2 ns).

### 2.4. Fluorescence (F_F_) from Water Samples Analysis

The F_F_ defined in Equation (1) is determined by the product between the absorbed light flux F_A_ and Φ_F_. Excitation energy for phytoplankton occurs on the Photosynthetically Active Radiation (PAR) spectral range, specifically 400–700 nm. F_A_, at a general depth z, can be calculated using the phytoplankton absorption coefficient ${\bar{a}}_{\mathrm{phy}}\left(z\right)$ spectrally weighted by the downwelling irradiance and the integral of the irradiance over the PAR, E_d_(PAR, z). All the information previously introduced and considering E_0_ as the spectral irradiance at the surface can be combined to rewrite Equation (1) as follow:

(3) $$F_{F}\left(z\right)=\Phi_{F}\left(z\right)*{\bar{a}}_{\mathrm{phy}}\left(z\right)*E_{d}\left(\mathrm{PAR},z\right)$$

in which ${\bar{a}}_{\mathrm{phy}}\left(z\right)$ Equation (4) and E_d_ (PAR,z) Equation (5) are defined, respectively, as:

(4) $${\bar{a}}_{\mathrm{phy}}\left(z\right)=\frac{\int_{400}^{700}a_{\mathrm{phy}}\left(\lambda ,z\right)E_{d}\left(\lambda ,z\right)d\lambda }{\int_{400}^{700}E_{d}\left(\lambda ,z\right)d\lambda }$$

(5) $$E_{d}\left(\mathrm{PAR},z\right)=\int_{400}^{700}E_{0}\left(\lambda ,0^{-}\right)e^{-k\left(\lambda \right)z}d\lambda$$

The final formulation of F_F_ used in the Lake Maggiore experiment is achieved re-writing F_A_ by means of Equations (4) and (5) obtaining:

(6) $$F_{A}\left(z\right)=\int_{400}^{700}a_{\mathrm{phy}}\left(\lambda ,z\right){\;E}_{0}\left(\lambda ,0^{-}\right)e^{-k\left(\lambda \right)z}d\lambda$$

and then:

(7) $$F_{F}\left(z\right)=\Phi_{F}\left(z\right)\int_{400}^{700}a_{\mathrm{phy}}\left(\lambda ,z\right)E_{0}\left(\lambda ,0^{-}\right)e^{-k\left(\lambda \right)z}d\lambda$$

where a_phy_ is measured in m^−1^, E_0_(λ,0^−^) represents the irradiance evaluated just below the water surface (in Wm^−2^ nm^−1^), while k is the coefficient accounting the light extinction with the water (in m^−1^).

All the terms displayed on the right side of Equation (7) have been measured during the sampling campaign (the 2 and 3 of July). In particular Φ_F_ and a_phy_ have been evaluated from the laboratory analysis described in Section 2.3, while the irradiance has been calculated exploiting the vertical water column profile carried out with the Satlantic radiometer. Since this instrument did not always reach the depths necessary for the experiment, i.e., z_SD_, the irradiance water column profiles have been exploited to retrieve first an experimental value of the attenuation factor k then used to calculate E_d_(λ,z_SD_). The experimental k has been obtained following the protocol described by Mishra et al. (2005) [51] assuming it is independent with the depth and considering the illumination on the water body stable during the single Satlantic acquisition, lasting in less than 10 min. For this reason, illumination correction factors have been not applied on the irradiances used. Integrating Equation (5) over the depth, instead of the PAR interval, and applying the approximations previously described, it was then possible to obtain an expression for k:

(8) $$-k\left(\lambda \right)*\left(z_{m}-z_{0}\right)=\ln\;\left(\frac{E_{d}\left(z_{m},\lambda \right)}{E_{d}\left(z_{0},\lambda \right)}\right)$$

where z_0_ represents the depth (in meters) closest to the surface (but below the water-air interface), z_m_ is chosen deeper than z_0_, while E_d_ (z_0_) and E_d_ (z_m_) correspond to the downwelling irradiances acquired by the Satlantic at z_0_ and z_m_, respectively. The k coefficient evaluated at a selected wavelength is obtained as the slope of the linear regression performed on the comparison between (z_m_ − z_0_) and the logarithm of the irradiance ratio showed on the right side of Equation (8). Since k has been assumed be independent with the depth, and therefore k(λ,z_m_) is equal to k(λ,z_SD_), the k spectrum over the PAR was obtained experimentally iterating the procedure for the different wavelengths in the spectral interval from 400 to 700 nm.

The E_d_ (PAR, z_0_) and E_d_ (PAR, z_SD_) have been calculated from Equation (5) and replaced in Equation (7) to evaluate the corresponding F_F_ values. Since Φ_F_ is unitless, a_phy_ is expressed in m^−1^ and E_d_ is an irradiance in Wm^−2^nm^−1^, it turns out the F_F_ is measured in Wm^−3^.

### 2.5. Carbon Fixation Proxy (Φ’_C_) from Water Samples Analysis

The procedure described in Section 2.4 can be also used to estimate F_C_ replacing the F_A_ explicit formula Equation (6) in Equation (2), and obtaining the following equation:

(9) $$F_{C}\left(z\right)=\Phi_{C}\left(z\right)\int_{400}^{700}a_{\mathrm{phy}}\left(\lambda ,z\right)\;E_{0}\left(\lambda ,0^{-}\right)e^{-k\left(\lambda \right)z}d\lambda$$

In contrast with the F_F_ case, in this equation two parameters are unknown, namely F_C_ and Φ_C_. Therefore, in the Lake Maggiore experiment, the F_C_ values have been replaced by the phytoplankton biovolume (in mm^3^ m^−3^), assuming that they are positively correlated to each other. This statement is agreement with previous studies demonstrating that the phytoplankton biomass (and then the biovolume) is the most important factor affecting the temporal variations of phytoplankton primary production [21,31]. Therefore, from Equation (8), it is possible to retrieve an experimental proxy for the quantum yield of carbon fixation Φ’_C_, defined as follows:

(10) $$\Phi_{C}^{\prime }\left(z\right)=\frac{\mathrm{biovolume}\;\left(z\right)}{\int_{400}^{700}a_{\mathrm{phy}}\left(\lambda ,z\right)E_{0}\left(\lambda ,0^{-}\right)e^{-k\left(\lambda \right)z}d\lambda }$$

calculated at z_0_ and z_SD_, respectively. Due to the approximations applied, Φ’_C_ is measured in mm^3^ W^−1^.

### 2.6. In-Situ Continuous and Hyperspectral Measurements

#### 2.6.1. Continuous Measurements Description

Lakes are characterized by fast phytoplankton dynamics, therefore a possible strategy to explore the inland waters complexity is by means of continuous and hyperspectral measurements acquired with a dense temporal resolution during the day and for consecutive days. In view of remote sensing applications, it is more useful working with the leaving water radiance L_w_, evaluated above the target instead of the L_u_, acquired in this case below the water surface. Therefore, to account for the water-air interface, the correction factor found in Zibordi et al. (2012) [52] has been multiplied to the L_u_ measured by the ROX. Specifically, this constant factor, equal to 0.543, accounting for the radiance decrease from below to above water surface is assumed to be wavelength independent according to Austin (1974) [53]. The L_w_ calculated with this approach has been divided by the E_d_, to obtain the remote sensing reflectance R_rs_. Since L_w_ is measured in Wm^−2^ nm^−1^ sr^−1^ and E_d_ in Wm^−2^ nm^−1^, the R_rs_ is reported in sr^−1^.

The ROX and Cyplops-7F times series have been preliminary merged to achieve acquisitions times coherent to each other. A data filtering has been then carried out considering several quality flags [54] that are automatically evaluated on the ROX spectral measurements. These quality flags allow detecting spectral measurements collected in not optimal illumination conditions, as it can occurs during cloudy conditions. Measurements that did not satisfied the quality flag imposed were deleted from the time series. All the continuous spectral and fluorometric measurements have been then averaged over a time interval of 10 min. The errors associated to them correspond to the standard deviations.

#### 2.6.2. Fluorescence Metric (F_FLH_) from Hyperspectral Measurements

The FLH method developed by Gower (1980) [55], is still frequently used today [10] as a valid approach to evaluate a proxy for the fluorescence emission in aquatic ecosystems. Briefly, it consists in a linear baseline derived by connecting two wavebands selected in the spectral ranges not affected by the fluorescence emission [56,57]. The F amount is evaluated subtracting the above-mentioned baseline from a selected central band, which is often placed close to the Chl-a fluorescent peak emission in the visible red (around 685 nm). The corresponding width, however, must exclude the absorption feature linked to the atmospheric oxygen band (O_2_B) at 687 nm [56,57]. This method is usually applied on the L_w_ and can be summarized in the two following equations:

(11) $$F_{\mathrm{FLH}}={\mathrm{Lw}}_{C}-{\mathrm{Lw}}_{\mathrm{baseline}}$$

(12) $${\mathrm{Lw}}_{\mathrm{baseline}}={\mathrm{Lw}}_{L}-\left({\mathrm{Lw}}_{R}-{\mathrm{Lw}}_{L}\right)*\frac{\lambda_{C}-\lambda_{R}}{\lambda_{L}-\lambda_{R}}$$

where λ_C_ is the fluorescence related central band, while λ_L_ and λ_R_ are the other two bands used for the virtual straight baseline, lying, respectively, on the left and on the right of λ_C_. In the equation above, L_wC_, L_wL_ and L_wR_ are the corresponding water leaving radiances. Particularly for Case-2 waters, the water body composition heavily influences the fluorescence estimations. For high Chl-a and TSM concentrations, indeed, the scattering dominates the signal acquired around 685 nm, as occur for example in trophic lake [16]. Conversely, under oligotrophic conditions (i.e., [Chl-a] lower than 3–5 mg m^−3^), the F signal in the red-NIR, linked to the chlorophyll pigments, is less affected by the residual absorption by CDOM and NAP (Non Algal Particles) [58,59]. Therefore, the assumption of a straight baseline to correct the F_FLH_ value for the scattering is valid [60]. For further information about this topic, please refer to [10]. Since the Lake Maggiore is characterized by low [Chl-a] and TSM concentrations (less than 3 mg m^−3^ and 1.50 mg L^−1^, respectively), the straight baseline approximation was applied to our case of study.

**Figure 2:**
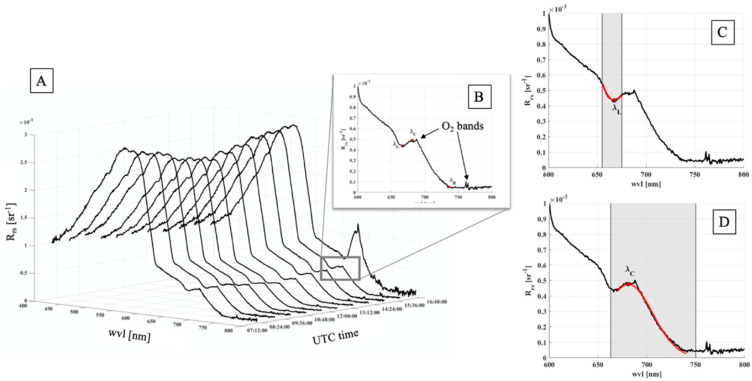
(**A**): times series corresponding to the R_rs_ spectra used to find the wavebands positions. It is clear a change in the spectra shape during the day, especially close to the sunset. (**B**): wavelengths range investigated. The two arrows highlight the artefacts due to the O_2_ absorption bands. Red points shown the positions of λ_C_, λ_L_ λ_R_, respectively. (**C**): the grey area shows the spectral range in which the λ_L_ has been searched. Red line represents the fit performed. (**D**): similarly, the λ_C_ has been evaluated by means of a gaussian fit (red line).

In the current work the waveband positions have been dynamically selected with respect the spectra changes occurring during the day to optimize the FLH algorithm. In this framework, the Rrs spectra have been exploited to carry out a preliminary research of the intervals in order to identify and standardize the wavelength positions (λ_C_, λ_L_, λ_R_) used in Equation (12). The method used to evaluate the λ_L_ and λ_C_ positions on the Rrs spectra is schematically represent in Figure 2. To remove the interference due to CDOM and NAP, λ_C_ should be as close as possible to λ_L_, but, at the same time, to be in a spectral region minimally affected by the Chl-a absorption. To minimize the backscattering contribution, the λ_R_ is chosen in a spectral region where pigments CDOM and NAP absorptions are negligible.

Summarizing, λ_L_ has been selected in the 655–675 nm spectral interval and corresponds to the R_rs_ minimum due to the Chl-a absorption (Figure 2C). Conversely, λ_C_ has been evaluated in the wavelengths range from 663 up to 750 nm. Even though the fluorescence contribution is maximum around 682 nm, a wide spectral window has been chosen in order to minimize the contribution of the absorption O_2_B band from the gaussian fit performed (Figure 2D). Finally, λ_R_ has been kept fix to 730 nm, because according to Kritten et al. (2020) [61], at this wavelength both the CDOM absorption and the NAP scattering are almost negligible.

This procedure has been applied on all the R_rs_ time series, but only the outcomes showing R^2^s greater than 0.70 have been considered. The remaining values, and corresponding spectra, have been deleted from the merged time series. The F_FLH_ have been evaluated on the L_w_ according to Equations (11) and (12), using the wavebands positions previously evaluated. In this framework, the corresponding bands widths have been calculated as the standard deviation of all the λ_L_ and λ_C_, respectively, while for the λ_R_ case, a nominal band width of 4 nm has been chosen.

#### 2.6.3. Spectral Indices from Hyperspectral Measurements

To predict the near-surface [Chl-a] in μg L^−1^ the Ocean Color (OCx) algorithm has been used. Conventionally, the OCx algorithm consist in a fourth-order polynomial relationship between a ratio of R_rs_ and [Chl-a]. The OCx approach was originally formulated to retrieve the [Chl-a] on mainly Case-1 waters. It relies on a ratio between the R_rs_ evaluated, respectively, in the blue spectral range, linked to the chlorophyll concentration, and in the green, less influenced by the chlorophyll contribution. According to the number of bands used, the OCx can be called OC_2_, OC_3_ and OC_4_, respectively. However, considering the characteristics of the Lake Maggiore described in Section 2.1, the low [Chl-a] and TSM concentrations (less than 3 μg L^−1^ and 1.50 mg L^−1^, respectively) and the CDOM value (0.04 m^−1^), the OCx rationale has been in first approximation applied also to optical complex waters (i.e., Case-2 waters). In this framework, the OC_4_ formulation has been used, in which the bands selected are 443 nm, 490 nm, 510 nm and 555 nm. The maximum band ratio (MBR) has been determined as the greater of the R_rs_(443)/R_rs_(555), R_rs_(490)/R_rs_(555) and R_rs_(510)/R_rs_(555) according with O’Reilly et al. 2000 [62]. Furthermore, the a_0_-a_4_ empirical regression coefficients used are 0.1731, −3.9630, −0.5620, 4.5008, −3.0020, respectively, Equation (13). In agreement with the nomenclature found in [62], the following equation has been used to evaluate the [Chl-a]:

(13) $${\left[\text{Chl-a}\right]}_{\mathrm{OC}4}=10^{\left(a_{0}+a_{1}R_{4}+a_{2}R_{4}^{2}+a_{3}R_{4}^{3}+a_{4}R_{4}^{4}\right)}$$

where:

(14) $$R_{4}=\log_{10}\left[\max\left({\mathrm{Rrs}}_{555}^{443},\;{\mathrm{Rrs}}_{555}^{490},{\mathrm{Rrs}}_{555}^{510}\right)\right]$$

To estimate the amount of light reaching the target, the E_PAR_, defined as the integral over 400–700 nm interval, has been evaluated exploiting the incoming irradiance (E_d_) measured by the ROX. Finally, the reflectance evaluated at 550 nm (R_550_) has been used as a proxy for the amount of light reflected by the water body.

### 2.7. Phytoplankton Primary Production Models

As shown in Equation (2), the carbon assimilation F_C_ due to the phytoplankton present in a unit of water volume is given by the product between the light absorbed by the chlorophyll pigments (F_A_) and the carbon fixation yield (Φ_C_). Following the approach proposed by Rossini et al. (2010) [23] for terrestrial vegetation, we tested different light use efficiency schemes by incorporating water spectral indices and fluorescence metric, as proxies for the conversion efficiency of energy to fixed carbon parameter Φ_C_ and F_A_. The new formulation for F_C_ by means of RS quantities is called in this work F_C-RS_. All the cases investigated and tested are summarized in Table 1.

In the first 5 cases (1–5), the Φ_C_ value is kept constant and its value is linked to the in-situ characteristic of the phytoplankton taxa. However, the Φ_C_ was not experimentally measured, therefore it has been replaced by the corresponding proxy Φ’_C_ evaluated as explained in Section 2.5. Since cases 1–5 assumed a constant carbon fixation yield, the average of all the Φ’_C_ available has been used and replaced in the corresponding F_C-RS_ formulations (Φ’_C_ = 331.69 mm^3^ W^−1^). Conversely, in cases from 6 to10, Φ_C_ could be linked to a non-constant yield accounting the dependence to the light availability and the phytoplankton status. Since the light availability affects the amount of light absorbed by the phytoplankton and the coupling between photosynthesis and fluorescence is known, this non-constant yield could be parametrized as the ratio between F_FLH_ and F_A_.

The parameter F_A_, is obtained by the product between the amount of light reaching the target (E_PAR_) and the phytoplankton absorption spectra a_phy_, according with its definition in Equation (6). E_PAR_ represents a parameter routinely measured or known in the remote sensing framework, while a_phy_ is usually obtained from in-situ acquisitions. By definition, a_phy_ depends on the chlorophyll-a concentration because it is given by the mean phytoplankton specific absorption coefficient a*_phy_ times [Chl-a]. Moreover, previous studies have highlighted a linear relationship between [Chl-a] and the phytoplankton primary production, demonstrating that the [Chl-a] is strongly positively correlated with phytoplankton primary production, as observed for instance by Deng et al. (2017) [21] in the Lake Taihu. For these reasons, F_A_ has been at first replaced by [Chl-a], and specifically by the spectral index [Chl-a]_OC4_ introduced in Section 2.6.3. Similarly, the amounts of light absorbed by the water body affects also the fluorescence emission and therefore F_FLH_ could be used as a proxy for F_A_. Finally, also combinations of [Chl-a]_OC4_, E_PAR_ and F_FLH_ have been investigated.

The F_C-RS_ parametrizations have been compared to the in-situ phytoplankton biovolume to assess the models robustness at local scale and for inland waters. Due to the low field measurements acquired and used in this work, only a preliminary analysis was possible.

## 3. Results

### 3.1. Characterization with the Water Samples Analysis

#### 3.1.1. Phytoplankton Composition

**Figure 3:**
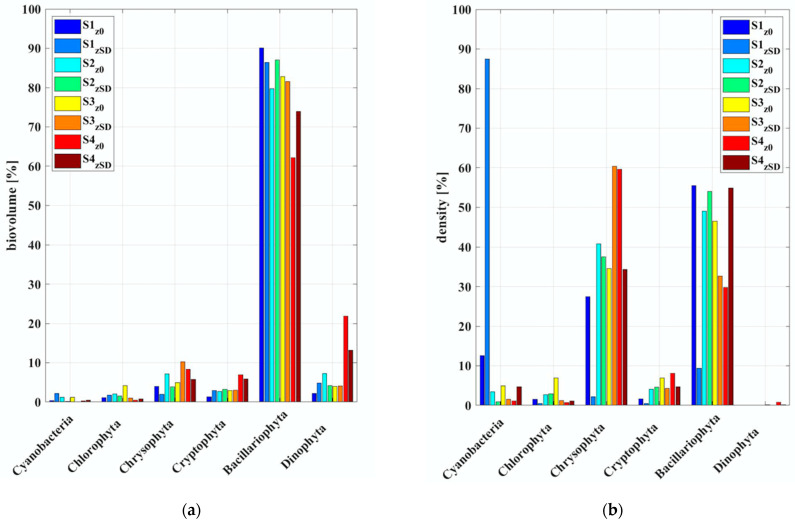
Composition of the phytoplankton major taxa expressed in percentages respect to biovolume (**a**) and density (**b**) in the water samples collected during the two-day water sampling. Colors indicate different samples depth and sampling dates.

Phytoplankton taxa composition in the water column was obtained by microscope counting of water samples collected at z_0_ and z_SD_, respectively. The taxa were identified to species level and counts converted to biovolume. Samples were dominated by Bacillariophyta (diatoms), with percentage above 60% of the total algal biovolume, for all the samples analyzed, regardless of the depth considered, the sample collection time and the meteorological conditions (Figure 3a). The 2 July, indeed, was characterized by clear sky conditions up to the solar noon then clouds appeared, while the 3 July was mostly cloudy.

If we consider the algal density instead of the biovolume (Figure 3b), the diatoms are still the dominant taxa in all the samples, with the exception of the deep sample collected on the morning of 2 July (S1z_SD_) where the most abundant class was the Cyanobacteria (*Aphanothece minutissima*), a group of non-vacuolate, small-celled and colonial with mucilage. The genera *Aphanocapsa*/*Aphanothece* are generally found in large colonies with cells characterized by very small biovolume compared with other taxa resulting in a low biovolume contribution despite relatively high cell concentrations and with a different content of chlorophyll-a. Moreover, mainly on the 3 July (S3 and S4), a codominance between Bacillariophyta and Chrysophyta (*Chrysochromulina* sp.) was observed.

#### 3.1.2. Phytoplankton Dynamics

**Figure 4:**
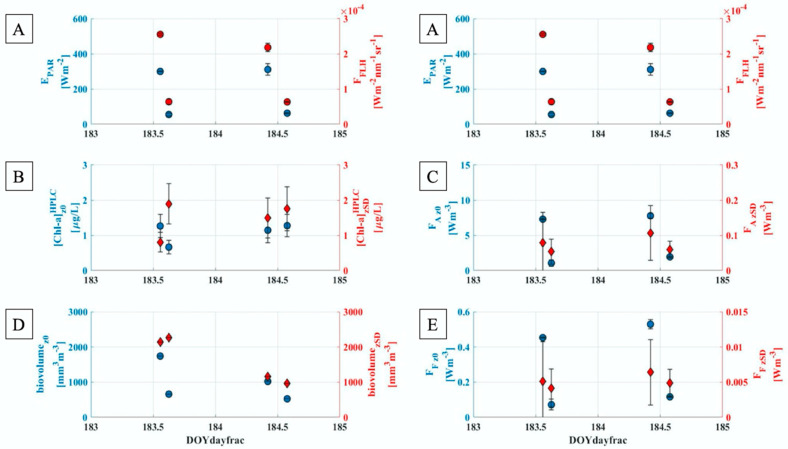
Dots represent variables measured close to the surface (z_0_), while the diamonds refer to those in the Secchi Disk depth (z_SD_). Time is shown as Day-Of-the Year (DOY): 183 is the 2 July 2019 while 184 is the 3 July 2019. First line ((**A**) left and right) shows the E_PAR_ (in blue) and F_FLH_ (in red) values in time. All the quantities displayed, except the biovolume (**D**), are mean values, where the error bars correspond to the standard deviations. Concerning [Chl-a]_HPLC_ (**B**) the averages have been carried out on the two replicas available. The F_A_ and F_F_ (**C**,**E**), instead, have been evaluated according Equations (6) and (7), in which the Satlantic irradiance spectra has been used. In these cases, each point displayed is the result of the average performed on two consecutive sets of measurements carried out. Furthermore, Satlantic acquisition times have been exploited to select the E_PAR_ and F_FLH_ from the ROX time series (**A**).

The aim of this section is to highlight the phytoplankton dynamics in different light conditions and at different depths. To achieve this goal, values obtained from the water samples, namely [Chl-a]_HPLC_ and algal biovolume, have been compared to F_A_ and F_F_, evaluated exploiting Equations (6) and (7), respectively. Moreover, the water samples collection time has been used to select from the hyperspectral and continuous time series the remote sensed parameters E_PAR_ and F_FLH_, linked to the amount of light reaching the target and the fluorescence emitted by a layer close to the surface, respectively. The evolutions of these values in time are displayed in Figure 4. The acronym DOY used hereafter and in Figure 4 stay for Day Of the Year. Specifically, 2 July corresponds to DOY 183, while the 3 July to DOY 184.

Figure 4 left panels show quantities obtained from the laboratory analysis (Figure 4B,D). Concerning the [Chl-a]_HPLC_, opposite diurnal trends have been observed at the surface (blue dots), according with the illumination and weather variability observed in the two consecutive days. In particular, under clear sky conditions, the amount [Chl-a]_HPLC_ is greater around the solar noon and then decreases together with the E_PAR_ (Figure 4A) (DOY 183). Conversely, with a predominant cloudy sky (DOY 184), the [Chl-a]_HPLC_ values are more stable throughout the day. The Secchi Disk depth (red diamonds), instead, seems less affected by the weather variability, with greater [Chl-a]_HPLC_ values from the solar noon up to the afternoon in both the two days investigated. Furthermore, the differences between DOY 183 [Chl-a]_HPLC_ values could be linked to the peak of Cyanobacteria (observed in the S1z_SD_ case), highlighted in Section 3.1.1. The biovolume, on the other hand, shows a completely opposite trend compared to the ones observed for the [Chl-a]_HPLC_, probably due to the different phytoplankton species present in the samples and not only characterized by the chlorophyll-a pigments. In general, in DOY 183 the biovolume values evaluated at the Secchi Disk depth are greater than the surface values, in which the difference between values evaluated around the solar noon and in the afternoon is clear. Conversely, during DOY 184, the biovolume shows similar values regardless of depth, acquisition times and illumination.

Figure 4 right panels, collect the evaluation of the amount of light absorbed by the water volume (F_A_) and the corresponding fluorescence emitted (F_F_) (Figure 4C,E). According to its parametrization, F_A_ is strictly linked to the irradiance reaching the target, indeed its diurnal evolution agrees with the one observed for the E_PAR_, regardless the depth considered. In particular, differences between surface and depth are restricted only to the discrete values, in which the light absorbed by the water body decreases with the depth. Similarly, F_F_ shows the same E_PAR_ (and then F_A_) diurnal evolution, also in agreement with the F_FLH_ trend. This last observation highlights the qualitative agreement between the in-situ and the remote sensed fluorescence. At the surface, there is a clear decrease of the F_F_ value from the solar noon to the afternoon in both the days investigated. Conversely, at the z_SD_, the fluorescence is less affected by the amount of the incoming light. In general, values linked to z_SD_ are characterized by a greater variability compared to the surface values, according to the standard deviations evaluated.

**Figure 5:**
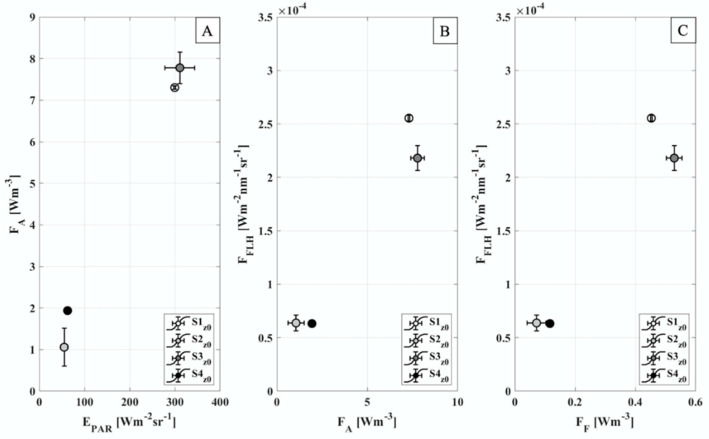
(**A**) shows the comparison between the E_PAR_ and the F_A_ evaluated with Equation (6); (**B**) the comparison between F_A_ and the fluorescence proxy evaluated with Equations (11) and (12); (**C**) shows the comparison between the fluorescence proxy F_FLH_ and the fluorescence evaluated from the water samples exploiting Equation (7). Values displayed corresponds to measurements evaluated at the surface. The colors (gray scale) help to discern between the several samples considered. All the measurements displayed here correspond to mean values, while the error bars to the standard deviations.

Interesting relationships between the water samples analysis outcomes and remote sensing quantities/indices have been observed in Figure 4. Nevertheless, a more detailed analysis has shown how this link is stronger when the z_0_ is considered. In particular, Figure 5 collects the meaningful trends that support the approximations introduced in Section 2.7 which are the basis of the several phytoplankton productivity models definitions. However, the few points used allow only a qualitative interpretation of the trends observed. Specifically, Figure 5A,B highlight a clear link between the light reaching the target (E_PAR_), the amount of light absorbed (F_A_) and re-emitted as fluorescence (F_FLH_). For this reason, both E_PAR_ and F_FLH_ could be used as a proxy for F_A_. Furthermore, the reliability of the novel FLH parametrization by means of dynamic waveband positions has been tested in Figure 5C. In this case, the F_FLH_ seems to be positively correlated to F_F_.

**Figure 6:**
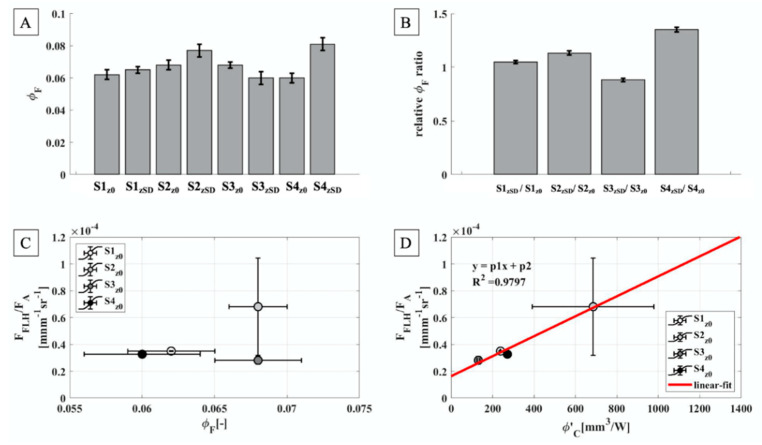
(**A**): Φ_F_ values obtained from the laboratory analysis referred on both z_0_ and z_SD_. (**B**): relative Φ_F_ ratio. Values linked to the Secchi Disk depth were divided by the corresponding surface values. (**C**,**D**): the comparison between the F_FLH_/F_A_ ratio with the Φ_F_ and Φ’_C_. Values displayed in the lower panels, correspond to measurements evaluated at the surface only. All the measurements displayed here correspond to mean values, while the error bars to the standard deviations. (**D**): S2_z0_ is characterized by a high uncertainty both on the x and y axes, probably due to the light variability during the measurements acquisition accounted the E_d_ term.

Eventually, a proxy for the fluorescence yield, retrieved from remote sensing quantities and defined as the ratio between F_FLH_ and F_A_ has been investigated (Figure 6). Under laboratory conditions, the maximal value of Φ_F_ obtained (i.e., when quenching due to photochemical photosynthetic processes is minimal) show only minor difference amongst the different samples collected (Figure 6A), regardless the depth considered. A relative Φ_F_ ratio has been also evaluated dividing the z_SD_ values by the z_0_ ones. In general, the ratio gives values close to or greater than one, with the exception of the S3 case which is characterized by decreased yield from the surface to the Secchi Disk depth (Figure 6B) and ratio value lower than one.

The laboratory Φ_F_ has been also compared to the fluorescence yield proxy (Figure 6C), however a clear link between these two parameters was not observed, probably because F_FLH_/F_A_ is derived from quantities that account a continuous excitation spectrum from 400 up to 700 nm, while Φ_F_ was obtained with an excitation wavelength centered at 632 nm. Moreover, the laboratory measuring conditions avoid artefacts due to re-absorption of emitted photons by the Chl-a pigments. Conversely, a very good correlation has been observed between F_FLH_/F_A_, which account a continuous excitation spectrum from 400 up to 700 nm (i.e., the whole PAR spectrum) and Φ’_C_ that is mainly obtained from field measurements (Figure 6D) with an R^2^ = 0.98.

### 3.2. Spectral Measurements Analysis

**Figure 7:**
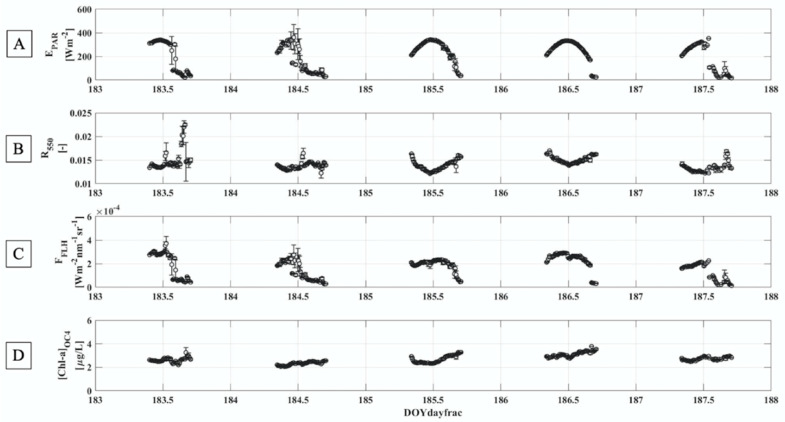
(**A**) shows the irradiance integrated over the PAR spectral range, between 400 and 700 nm; (**B**) shows the reflectance evaluate at 550 nm; (**C**) shows the fluorescence proxy obtained with the dynamically waveband FLH approach; (**D**) displays the spectral index linked to the chlorophyll-a concentration. Data shown represent the mean values (n_max_ per interval ~10), averaged on a time interval of 10 min, while the error bars correspond to the standard deviations.

The week-long times series acquired by means of the ROX and the Cyplops-7F sensors has been studied to characterize the phytoplankton diurnal dynamics with a hyperspectral and temporal dense resolution. Figure 7 shows selected spectral quantities related to the phytoplankton behavior. In particular, E_PAR_ indicates the amount of light reaching the target (Figure 7A). DOY 185 and DOY 186 are characterized by the trend typical of the clear sky days (E_PAR_~ cos(SZA)). The small standard deviation values indicate stable illumination conditions during the time interval averaged (10 min). Conversely, a larger variability is observed during DOY 184, characterized by clouds especially in the afternoon. DOY 183 and DOY 187, instead, show mixed sky conditions, in particular a drastic drop of the light intensity is observed after the solar noon.

The reflectance evaluated at 550 nm represents the portion of light reflected by the surface (Figure 7B). In the two clear sky days identified, a minimum is reached in correspondence with the E_PAR_ maximum, while the R_550_ diurnal evolution in cloud sky conditions is more variable, probably because of a greater contribution from diffuse with respect to the direct light reaching the detector.

F_FLH_ (Figure 7C) is a proxy for fluorescence and its daily trend is comparable to the one observed for the E_PAR_. Furthermore, DOY 185 and DOY 186 showed a peculiar behavior around the solar noon. The solar noon occurs when the shortest path of light in the atmosphere is reached, therefore the amount of energy available to be absorbed by the target is greater compared to the other hours and could then affect the fluorescence emission. This time window is interesting to be investigate because it is characterized by the maximum of the E_PAR_, the R_550_ minimum and the F_FLH_ local minimum.

**Figure 8:**
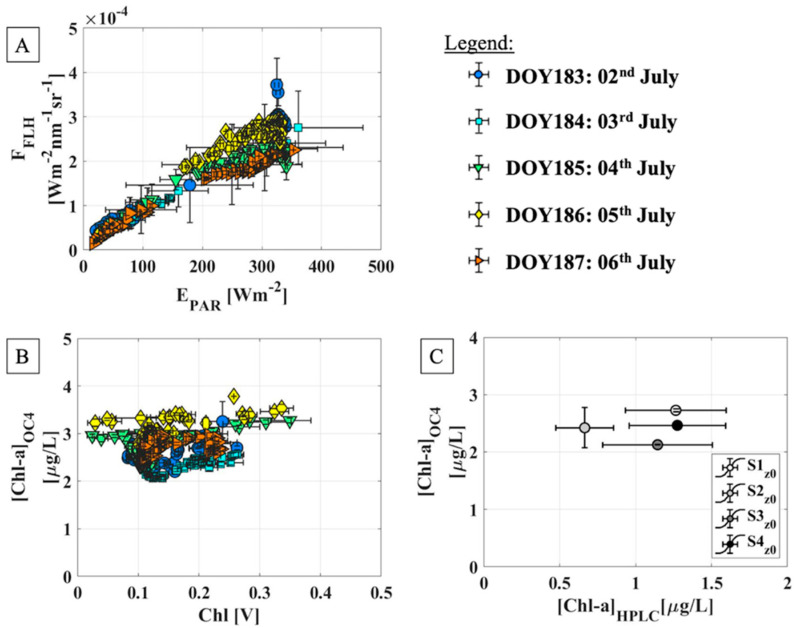
(**A**) shows the comparison between E_PAR_ and F_FLH_ throughout the time series. Each days of observation is characterized by different symbols and colors displayed in the legend on the top right. (**B**) collects the two chlorophyll concentration parameters: on the x axes there are the fluorometer values (in Volt), while on the y axes the spectral index is evaluated from the reflectance exploiting the OC_4_ approach. Furthermore, (**C**) shows the comparison between the [Chl-a] values obtained from the laboratory analysis and the spectral measurements.

The [Chl-a]_OC4_ has been used to evaluate the chlorophyll-a concentration (Figure 7D). The algorithm should give as outcome of the [Chl-a] in µg/L. However, the results obtained overestimate the in-situ values (see also Figure 8C). This mismatch is probably due to the regression coefficients (a_0_–a_4_) used in the Equation (13) that are not optimized for this specific water body. Nevertheless, a qualitative analysis has been carried out. Almost all the days investigated are characterized by a diurnal, monotonic, growth of this index. Again, the two clear sky days showed a peculiar trend around the solar noon where a minimum is reached.

The hyperspectral measurements and indices acquired and calculated from the time series have been also exploited in the phytoplankton model’s definition (Figure 8). In particular, a linear relationship between the E_PAR_ and the F_FLH_ is clear for all the days of the experiment (Figure 8A) and this pattern was also observed in the terrestrial vegetation, when the E_PAR_ is compared to the fluorescence evaluated at 760 nm.

Concerning the [Chl-a], the OC_4_ method reliability has been investigated by means of the chlorophyll measurements collected with the fluorometer (Figure 8B). Globally, there is not a clear linear correlation between Chl [V] and [Chl-a]_OC4_, probably because the OC_4_ method was developed specifically for the chlorophyll-a pigments only. Nevertheless, the [Chl-a]_OC4_ seems to be related to the in-situ [Chl-a]_HPLC_ even though a robust positive correlation is not clear due to the low values available (Figure 8C).

### 3.3. Phytoplankton Primary Production Models Test

Considering all the relations highlighted in Section 3.1.2 and Section 3.2, the models summarized in Table 1 have been tested. Since only four points, corresponding to the surface samples, were available, the analysis carried out were mainly qualitative. A numerical comparison was not possible because the approximations taken. Specifically: (i) the constant used as in-situ value in Cases from 1 up to 5 is a proxy for Φ_c_; (ii) the biovolume has been used instead of F_C_ to validate the models; (iii) the [Chl-a]_OC4_ overestimates the true field values and (iv) the F_FLH_ is a proxy for F_F_.

**Figure 9:**
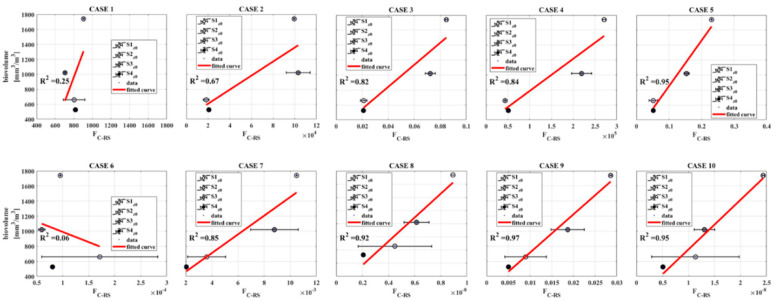
The first row shows the cases in which the Φ_C_ proxy has been kept constant, while the F_A_ has been replaced by hyperspectral measurements and indices. Second row shows the cases in which also the Φ_C_ has been replaced by a proxy defined from remote sensed quantities. The red lines correspond to the linear regression performed on the measurements. The scale for F_C-RS_ have been omitted on purpose because due to the approximations taken only a qualitative comparison was possible.

Considering all these approximations, an exploratory analysis has been carried out and the results obtained are shown in Figure 9. When only the spectral index for [Chl-a]_OC4_ is used to replace the portion of absorbed light (namely F_A_), the model fails, regardless of the Φ_C_ parametrizations (Case 1 and Case 6). Conversely, when F_A_ is replaced by E_PAR_, the models describe better the field behavior, with the R^2^, equal to 0.67 and 0.85 for Case 2 and Case 7, respectively. The statistics improve when the F_FLH_ is used, because this parameter is strictly linked to the amount of light actually absorbed by the Chl-a pigments. In this case R^2^ greater than 0.80 have been observed in Case 3 and 8. When F_A_ is parametrized with two terms, one linked to [Chl-a] and the other one to the available light, the parametrization of Φ_C_ plays a key role. When Φ_C_ is kept constant, the Case 5, in which both [Chl-a]_OC4_ and F_FLH_ are used, shows a very good correlation statistic corresponding to an a R^2^ of 0.95. Further improvement is obtained when Φ_C_ is replaced by a proxy of the fluorescence yield. In general, the Case 9 parametrization gives the highest performance with an R^2^ equal 0.97.

## 4. Discussion

All the quantities processed and studied in this work are referred to the Lake Maggiore case study and have been collected during a field campaign carried out in July 2019. The lake water has been characterized by phytoplankton microscopic counting. In terms of biovolume percentage, in all the samples analyzed it is clear a dominance by the Bacillariophyta (diatoms). This result agrees with the observations previously reported in Morabito et al. (2007) [63], in which the Spring cluster (from April to mid-July) was characterized by the Bacillariophyta, specifically the most abundant species was the *Fragilaria crotonensis*, and in Morabito et al. (2002) [64] where large pennate such as *Fragilaria crotonensis* characterized the phytoplankton summer composition in Lake Maggiore. This is important because diatoms are frequently the key component of phytoplankton assemblages [65]. They are responsible for 20 to 25% of global carbon fixation [66] and serve as the basis for pelagic food webs [67].

In general, the diatom cells are narrower and contained more chlorophyll. In particular, the Fragilaria growth is constrained by the light availability and the cell number of its colonies are lower than at the surface, as reported in the parallel study [68]. In the Lake Maggiore experiment, a decreasing trend of the Bacillariophyta biovolume and density from surface (z_0_) to depth (z_SD_) was observed in the water samples collected around the solar noon, namely S1 and S3 (Figure 3). Conversely, an opposite trend was observed in samples collected in the afternoon (S2 and S4) with greater values at z_SD_. These results support the strong link between the Bacillariophyta (and then the Fragilaria) stratification in the water column driven by the light availability. According to Reynolds (1997) [69] the increase of cell number into a colony decreases the sinking rate. Similarly, Morabito et al. (2003) [70] asses that the decrease of cell number is the results of the need of contrasting the sinking and making the colony lighter in a stratified water column during summer. Further, as reported in Reynolds (1997) [69], the elongated shape of the diatom *Fragilaria crotonensis* makes these algae very efficient in utilizing the available underwater PAR, giving them a competitive advantage at low light intensities and are very sensible to water column stratification [71].

Finally, a peak of Cyanobacteria has been observed at z_SD_ the 2 July, in the sample collected close to the solar noon (S1z_SD_). This anomalous value could be linked to the copious rainfalls registered on the 1st of July, in the afternoon, affecting the Cyanobacteria growth.

Since Case-2 waters are very optically complex and undergo sudden changes at diurnal scale, further analysis were carried out. From the water samples, the two indicators exploited to follow the phytoplankton evolution in time were the [Chl-a]_HPLC_ and the biovolume, the latter one used as a proxy for the biomass. Moreover, the a_phy_ and Φ_F_ (from laboratory) and irradiances spectra (collected by the Satlantic) were replaced in Equations (6) and (7) to evaluate the in-situ F_A_ and F_F_, respectively. All the parameters selected were investigated at two specific depths, z_0_ and z_SD_, to characterize the phytoplankton dynamics also within the water column. Conversely, hyperspectral and continuous measurements, acquired by the ROX and only referred to the water surface, were analyzed to calculate the remote sensing metric linked to the fluorescence (F_FLH_) and the E_PAR_, the latter one used as reference to account the different illumination conditions. The qualitative comparison is shown in Figure 4.

Concerning the surface, the [Chl-a]_HPLC_ shows a clear dependence on the weather variability, with diurnal opposite trends observed in the two consecutive days observed. We remind, indeed, that the 2 July (DOY 183) was characterized by clear sky conditions up to the solar noon then clouds appeared, while the 3 July (DOY 184) was mostly cloudy. The biovolume, instead, decreases with the E_PAR_ regardless of the day considered. Conversely, at z_SD_ the [Chl-a]_HPLC_ shows a common path with lower values close to the solar noon in both the two days investigated, while the biovolume is almost constant during the day. Focusing on Figure 4B,C, generally samples S2, S3 and S4 exhibit values slightly greater at the z_SD_ depth (diamonds) respect to the z_0_ ones (blue dots) excepted for the S1 case. However, this anomalous outcome can be explained to the peak of Cyanobacteria observed in the sample S1z_SD_.

F_A_ and F_F_, evaluated at the surface (Figure 4C,E), show temporal evolutions in agreement with the ones highlighted for E_PAR_ and F_FLH_. Specifically, F_A_ seems to be positively related to both the amount of light reaching the target (E_PAR_) and the energy dissipated as fluorescence (F_FLH_) (Figure 5A,B). Similarly, F_F_ exhibits a linear trend with F_FLH_ (Figure 5C). The qualitatively comparisons highlighted in Figure 5 agree with an invariance of the maximal yield of Chl-a fluorescence emission (Φ_F_), statement also supported by the laboratory analysis (Figure 6A). Largely invariant value of Φ_F_ agrees with the good correlation between the F signal retrieved in-situ and the phytoplankton concentration, as well as the E_PAR_. Actually, strong variation in the Φ_F_ which could be due to quenching process of non-photochemical nature, are expected to lead to breakdown a linear correlation between species concentration and monitored fluorescence levels. Given the relatively small differences in terms of Φ_F_ in all the samples analyzed, it is possible to assess that in the days investigated (DOY 183 and DOY 184) the photochemical quenching was almost negligible. However, the relative Φ_F_ ratio obtained dividing the z_SD_ values by the corresponding z_0_ ones (Figure 6B) showed values closer/greater than one, excluding the S3 case. Therefore, for all the samples, except for S3, the surface values appear weakly quenched compared to the ones evaluated at z_SD_.

A proxy for the fluorescence yield has been then defined as the ratio between F_FLH_ and F_A_ and compared to the laboratory Φ_F_ values and to the experimental proxy for the quantum yield of carbon fixation (Φ’_C_) (Figure 6B,C, respectively). While the two fluorescence parameters do not show a clear connection to each other, a linear correlation is instead observed between Φ’_C_ and F_FLH_/F_A_, possibly due to their respective parametrizations. These last two terms, indeed, have been evaluated exploiting field measurements considering the whole PAR spectral interval.

In agreement with this observation, one of the goals of this work was to assess whether hyperspectral and temporal dense remote sensed measurements are able to follow the phytoplankton diurnal dynamics characteristics of inland waters, overcoming the limits represented by in-situ sampling and laboratory analysis bias. High frequency investigations have been carried out, where E_PAR_, R_550,_ F_FLH_ and [Chl-a]_OC4_ have been used to investigate and characterize the phytoplankton behavior at local scale. In particular, a novel FLH algorithm optimized for optically complex waters here developed and has been used.

A peculiar trend has been observed around the solar noon in the two clear sky days identified according to the E_PAR_ shape (DOY 185 and DOY 186): when the E_PAR_ reaches the maximum, both the F_FLH_ and the [Chl-a]_OC4_ drop (Figure 7). The most likely explanation of the fluorescence decrease around the solar noon is the occurrence of non-photochemical quenching of the fluorescence under very bright natural light, which can either be linked to regulative, or more likely, light-induced stress (given that the quenching is conserved and stable in the sample, whereas regulative quenching relaxes rapidly (e.g., [72,73,74]).On the other hand, according to Reynolds et al. (2006) [75], the phytoplankton act strategies to escape the harmful photoinhibition caused by oxidative stress of excessive insolation near the top of the water column consequently have the effect of cutting photosynthetic rate and vertical migration exist. This last hypothesis agrees with the diurnal [Chl-a]_OC4_ trend (Figure 7D). Focusing on the clear sky days, around the solar noon lower [Chl-a]_OC4_ values are reached. Since this spectral index is evaluated at wavelengths not influenced by the fluorescence emission, this behavior can be ascribable to a shift of the phytoplankton organisms deeper in the water column rather than to non-photochemical quenching. However, Figure 6B highlighted a weak surface quenching. It is hence likely that both movement of the phytoplankton through the water column and changes Φ_F_ accounts for the experimental observables.

The continuous spectral measurements were also exploited to evaluate the link between the amount of light reaching the target (E_PAR_) and the F_FLH_ metric. The linear relation observed for all the days composing the time series supports again the hypothesis of an almost invariant Φ_F_ (Figure 8A). Moreover, the F_FLH_ could be used as a proxy for the incoming irradiance. At diurnal scale, values corresponding to clear sky days (DOY 185 and DOY 186) are gathered in a restricted portion of the plot as the illumination changes slowly during the day and then the phytoplankton adaptivity is gradual. Conversely, when the light available oscillates due to the cloud presence, extreme situations could be reached. Therefore, also very low E_PAR_ and F_FLH_ values could be observed because the phytoplankton undergoes sudden and rapid illumination changes. However, it is worth to note that the above-mentioned linear trend is observed for all the day long, except for the points corresponding to high E_PAR_ values and then to the solar noon. Interestingly, this is the only environmental condition under which a weak decrease in Φ_F_ (referred to the surface) was assessed, likely associated from slowly reversible non-photochemical fluorescence quenching. Thus, the decrease in Φ_F_ could contribute to apparent discrepancy, together with phytoplankton migration, observed under very bright environmental lights. Furthermore, trends displayed in Figure 8A supports the reliability of the new FLH parametrization by means of dynamically chosen wavebands, because in the terrestrial vegetation a similar diurnal trend is observed when the E_PAR_ is compared to the fluorescence evaluated at 760 nm.

Primary production by phytoplankton is a fundamental process underlying lake metabolism [76] and the knowledge of the spatial variations of the primary production, nutrient concentration and community structure is fundamental to the understanding of ecosystem dynamics [77]. Nowadays, in inland water, the phytoplankton primary production is mainly obtained by in-situ analysis that restricts the spatio-temporal information. However, with the advent of novel hyperspectral instruments this limit could be overcome, in particular in view of the upcoming FLEX (FLuorescence EXplorer) mission. This satellite mission is currently under preparation by the European Space Agency (ESA) and planned to be launched in 2024. The five-year global mission, completely dedicated to vegetation fluorescence measurements with a spectral resolution of 0.3 nm in the VIS-NIR, will cover both terrestrial and aquatic ecosystems, producing imagery and maps with a 300 × 300 m^2^ spatial resolution [78].

Results from Section 3.1 and Section 3.2 have clearly demonstrated, from one side, the strong link between in-situ and remote sensed measurements, and from the other, how the hyperspectral resolution is suitable to follow better the phytoplankton temporal dynamics.

For this reason, remote sensed measurements and indices have been opportunely combined to define a remote sensing PP proxy (F_C-RS_) suitable for lake ecosystems, therefore phytoplankton PP models have been tested. In particular, the validity has been assessed comparing the different F_C-RS_ parametrizations with the biovolume. Since only few field measurements were available for this test, the preliminary analysis carried out was mainly qualitative and restricted to local scale. The correlations highlighted do not have statistical meaning, but they have been used only to asses which F_C-RS_ formulation better follow the biovolume evolution in time.

When the [Chl-a]_OC4_ is replaced to the F_A_, the F_C-RS_ obtained are not correlated to the biovolume, regardless the Φ_C_ parametrization adopted (Figure 9, Case 1 and Case 6). This outcome is, in first approximation, in contrast with what was assessed in Deng et al. (2017) [21]. However, it is better to remark that in Deng et al. (2017) [21] the biomass has been compared to the [Chl-a], while in the Lake Maggiore case, the biovolume and a spectral index were used instead. Nevertheless, the model failure in both Case 1 and Case 6 trends could mean that the [Chl-a]_OC4_ alone is not enough to describe the F_A_ term and then the biovolume diurnal evolution. Conversely, promising results have been reached when the F_A_ was replaced first by the E_PAR_, Case 2 and 7, with R^2^ of 0.67 and 0.85, respectively. The statistics improve further when the F_FLH_ is considered, where R^2^ greater than 0.80 (Case 3 and 8), probably because the F_FLH_ depends intrinsically to the phytoplankton concentration and then is more strictly linked to the biovolume. Therefore, the coupling between fluorescence and photosynthesis could be exploited to obtain a reliable phytoplankton productivity model. We remark that these outcomes agree with the study by Barnes et al. (2014) [79], in which is highlighted that the rate of phytoplankton primary production is primarily a function of the incident irradiance (E_PAR_), light absorption efficiency (linked to F_FLH_) and the quantum efficiency of carbon fixation (Φ’_C_ from field values or the spectral proxy F_FLH_/F_A_).

Finally, a brief consideration about the chlorophyll-a parameter. The [Chl-a] is the main driver of variability in primary production in the global ocean and thus simple empirical relationships that directly relate [Chl-a] to primary production have been used in marine sciences. However, the existing models developed for ocean waters are not suitable for lakes [76]. Indeed, the parametrizations used in Cases 1 and 6 fail when only the [Chl-a]_OC4_ is considered. Conversely, when the [Chl-a]_OC4_ is coupled with the E_PAR_ and F_FLH_, respectively, the statistics improves. In this context, the F_C-RS_ parametrization that gives the best performance is the one developed for the Case 9 (R^2^ = 0.97). Here, the Φ_C_ has been chosen to be non-constant in order to account the dependence to the light availability and the phytoplankton status. It was replaced by the ratio between F_FLH_ and F_A_, linked to Φ’_C_, shown in Figure 6D. Finally, F_A_ was obtained by the product between E_PAR_ and [Chl-a]_OC4_, the latter one used as proxy for a_phy_, consistently with the F_A_ definition showed in Equation (6).

## 5. Conclusions

This preliminary study has demonstrated how the hyperspectral and temporal resolutions are suitable to follow the phytoplankton dynamics, particularly in clear sky days. The spectral indices calculated are strictly linked to lake water characteristics, measured in laboratory. Furthermore, several phytoplankton primary production models driven from remotely sensed data have been tested on the Lake Maggiore. The preliminary outcomes obtained demonstrated that, at local scale, remote sensing represents a sensitive tool for monitoring temporal variations in phytoplankton PP in lakes, as also observed in previous study. Here, we originally introduce the fluorescence yield as a proxy of the light use efficiency parameter. In general, the low number of points used to test the several PP models do not allow us to assess which phytoplankton productivity model is correct for the Lake Maggiore case, but this preliminary analysis highlighted how the statistics improve when the F_FLH_ is replaced instead of F_A_. In summary, it seems that Case 5 and Case 9 are the best models for inferring productivity in our study area and particularly when fluorescence yield is used as a proxy of carbon fixation efficiency, similarly to what found in terrestrial ecosystems. This cannot be probably extended to other lakes and further investigations and validations are needed. Even though it represents only a preliminary study carried out at local scale, the future goal is to exploit RS fluorescence to retrieve the phytoplankton PP in order to overcome the limits related to the sparse measurements of typical in-situ sampling and improve the inland waters spatio-temporal monitoring and understanding.

## Figures and Tables

**Table 1:** List of all the F_C-RS_ parametrizations investigated and tested in this work.

CASE ID	Φ_C_	F_A_	CASE ID	Φ_C_	F_A_
1	constant	[Chl-a]_OC4_	6	F_FLH_/F_A_	[Chl-a]_OC4_
2	constant	E_PAR_	7	F_FLH_/F_A_	E_PAR_
3	constant	F_FLH_	8	F_FLH_/F_A_	F_FLH_
4	constant	[Chl-a]_OC4_·E_PAR_	9	F_FLH_/F_A_	[Chl-a]_OC4_·E_PAR_
5	constant	Chl-a]_OC4_·F_FLH_	10	F_FLH_/F_A_	Chl-a]_OC4_·F_FLH_

## Data Availability

The datasets generated and/or analyzed during the current study are available from the corresponding author on reasonable request.
